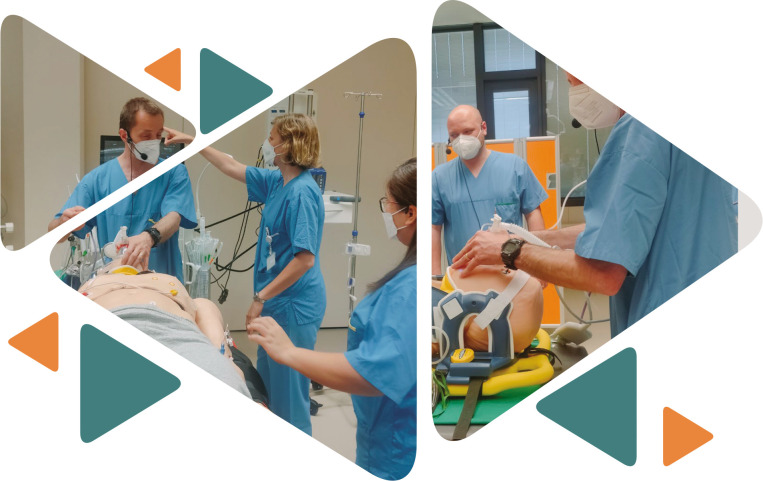# Neurotrauma Treatment Simulation Center (NTSC) Vienna – Event Report

**DOI:** 10.25122/jml-2022-1015

**Published:** 2022-07

**Authors:** Andreea Doria Constantinescu, Stefana-Andrada Dobran, Diana Chira

**Affiliations:** 1. RoNeuro Institute for Neurological Research and Diagnostic, Cluj-Napoca, Romania; 2. Sociology Department, Babes-Bolyai University, Cluj-Napoca, Romania

## AMN (ACADEMY OF MULTIDISCIPLINARY NEUROTRAUMATOLOGY)

The Academy for Multidisciplinary Neurotraumatology (AMN) represents a promising scientific organization aiming to advance research, practical application, and education in neu- rotraumatology.

It organizes international congresses, facilitates communication between national and international societies, as well as academies and associations with a common interest in neurotrauma, and develops innovative educational programs-including the Neurotrauma Treatment Simulation Center (NTSC).

The AMN is a modern scientific society with members from a wide-range of fields (*e.g*. neurology, neurosurgery, rehabilitation, psychology, psychiatry, *etc*.), from all over the world, focusing on the importance of long-term follow-up after neurotrauma, and promoting the concept of multidisciplinary treatment.

With over 600 members, as well as partnerships with the European Federation of NeuroRehabilitation Societies, World Federation for Neurorehabilitation, European Society of Clinical Neuropharmacology, Foundation of the Society for the Study of Neuroprotection and Neuroplasticity, RoNeuro Institute for Neurological Research and Diagnostic, Journal of Medicine and Life, Foundation for the Study of Nanoneurosciences and Neuroregeneration, Neurotech^EU^, the AMN is on the path of becoming a dynamic force strengthening state-of-the-art neurotrauma treatment globally.

One of the key developments of the AMN is PRESENT (Patient REgistry – Short Essential NeuroTrauma), discussed in the next pages, representing an innovative approach to Neurotrauma Management which can be used worldwide. This TBI registry offers a technological solution to improve information collection on TBI epidemiology and quality indicators at the national and international levels.

Ultimately, the registry aims to collect sufficient clinical research data and enhance the understanding of the patient's recovery pathway in different medical systems.

## THE ROLE OF PRESENT

During the Neurotrauma Treatment Simulation Center (NTSC) week in Vienna, each of the six participating groups pointed out, in their country presentation, that one of the most important problems that they are confronted with is the lack of a traumatic brain injury (TBI) registry.

This is the main reason why the Academy for Multidisciplinary Neurotraumatology (AMN) develops PRESENT. Its aim is to support doctors globally, in their mission to collect longitudinal data from all care levels of TBI, and to serve as a go-to hub for basic TBI data collection in all countries, both in those with low access to data surveillance mechanisms, as well as in countries with a more advanced healthcare system.

The registry's multidisciplinary approach becomes apparent by the fact that all specialities involved in the treatment of TBI (emergency staff, neurologists, neurosurgeons, Intensive Care Unit (ICU) specialists and rehabilitation specialists) will be able to include the most relevant data throughout the continuum of the patient's recovery process-from ICU to completing rehabilitation. The focus on a multidimensional approach ensures the measurement of the true standard of care and helps generate performance improvement indicators.

The questionnaire can be filled out by each physician within only 10 minutes, which should add motivation to complete the forms.

PRESENT will help obtain worldwide statistics on TBI and use these data to improve treatment and create a standard of TBI care that could be implemented everywhere in the world.

## INTRODUCTION

The Neurotrauma Treatment Simulation Center (NTSC) is a unique educational program conducted in its' first edition in Vienna, Austria, between May 16^th^ and 20^th^ in 2022 with the aim to promote multidisciplinary neurotrauma treatment concepts. The five-day training followed the patient pathway, from emergency care up to rehabilitation, and brought together specialists with a common interest in the management of neurotrauma and diverse backgrounds (*i.e*., neurology, neurosurgery, neurocritical care, anesthesiology, traumatology), from six countries (Romania, Poland, Mexic, Slovakia, Egypt, and the Philippines). The full list of participants can be accessed here.

The training aimed to identify how to change the treatment paradigm in neurotraumatology from short-term focus to long-term follow-up. Moreover, of central importance for NTSC has been to promote a multidisciplinary treatment concept throughout the chain of recovery and to discuss methods to overcome the lack of collaboration among medical specialists involved in this chain.

Other important objectives of NTSC were that the multidisciplinary teams from all six countries develop a mutual understanding of the complex needs of Traumatic Brain Injury (TBI) patients at each stage of treatment and reduce the treatment nihilism about cognitive, behavioral, and depressive disorders in acute care.

The delegates were given the opportunity to discuss, suggest, and agree on the most significant quality indicators of care, which would be measured in a new TBI registry (PRESENT). Ultimately, the goal of each country's delegation was to construct a plan on how multidisciplinary teams could be developed in their own countries.

This innovative event was organized by The Academy of Multidisciplinary Neurotraumatology together with its academic partners: RoNeuro Institute for Neurological Research and Diagnostic, Journal of Medicine and Life, The Society for the Study of Neuroprotection and Neuroplasticity and Neurotech^EU^.

The organization of the event would not have been possible without the support of the distinguished faculty members of NTSC Vienna, who built up the program and coordinated the very complex 5 day-training. The lectures of the faculty members showcased the treatment and research of neurotrauma under different aspects and from different perspectives, depending on their specialities, all highlighting the importance of a multidisciplinary approach and the need for a long-term follow-up of the patients.

This educational and practical event has been set up to un-fold over 5 days in four different locations:

- Allgemeines Krankenhaus Wien (AKH) on Day 1 (Monday) and Day 3 (Wednesday);
- Landesklinikum Wiener Neustadt on Day 2 (Tuesday);
- Klinik Pirawarth on Day 4 (Thursday);
- Klinik Floridsdorf on Day 5 (Friday).

The full program of NTSC can be found here.

A total of 18 delegates from the six countries – Egypt, Mexico, the Philippines, Poland, Romania, and Slovakia – participated in the intensive training under the coordination of five main tutors:

- Christian Matula – Professor and Vice-Chairman, Department of Neurosurgery, Allgemeines Krankenhaus Wien (AKH);
- Johannes Leitgeb – Assoc. Prof. Neurosurgery, Allgemeines Krankenhaus Wien (AKH);
- Andreas Winkler – Medical Director, Head of Neurological Rehabilitation Department, Klinik Pirawarth;
- Peter Lackner – Head of Department of Neurology at Klinik Floridsdorf;
- Helmut Trimmel – Professor, Director of the Department Anaesthesiology, Emergency and Critical Care Medicine Landesklinikum Wiener Neustadt (represented on-site by Dr. Daniel Csomor and Dr. Gunther Herzer).

The Academy of Multidisciplinary Neurotraumatology has been represented by its President, Professor Johannes Vester.

All participants are specialists who are normally part in the treatment chain of neurotrauma. Their relevant experience and wish to gain new skills and knowledge in their daily practice were the main reasons for being part of this training. The dedication and involvement of the tutors and delegates made the NTSC program a unique and insightful experience.

**Figure:**
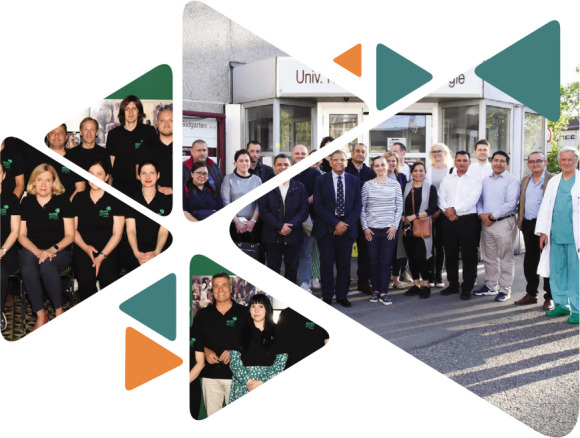


## INTERVIEWS

As an essential part of the program and for the future development of similar projects, the AMN coordinating team scheduled interviews with all country delegates and program coordinators. Interviews with delegates ran in two distinct sessions: (1) *pre-event interviews* and (2) *post-event interviews*. The former focused on the presentation of each delegate along with their insight on a variety of topics (*e.g*., differences in approach to neurotrauma care and research, country-specific issues, limitations on the management of neurotrauma, the various perspectives on the need for an international registry, multidisciplinary teams, and online communities). The latter showcased the delegates' feedback on the event and potential activities for future similar trainings.

The interviews with the faculty focused on their motivation to lead the NTSC training, participation in any similar programs implemented to date, on identifying the limitations in the approach to cognitive and behavioral disorders resulting from neurotrauma, the most pressing organizational issues in the treatment and rehabilitation of neurotrauma, and obstacles that affect a proper long-term follow-up of patients. The interviews are highlighted on the AMN Blog.

**Figure:**
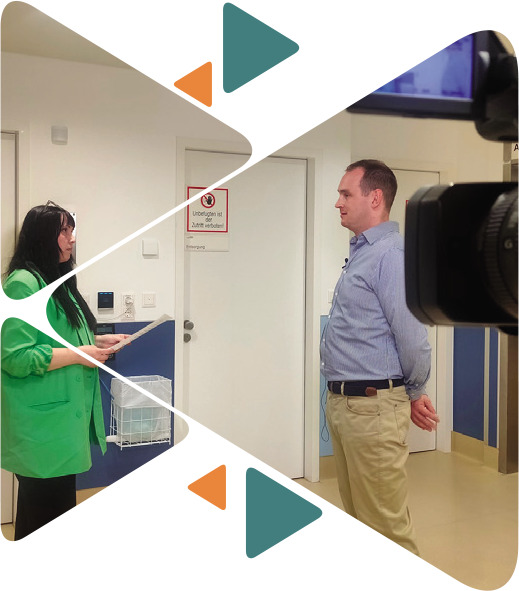


## FACULTY MEMBERS

**Figure:**
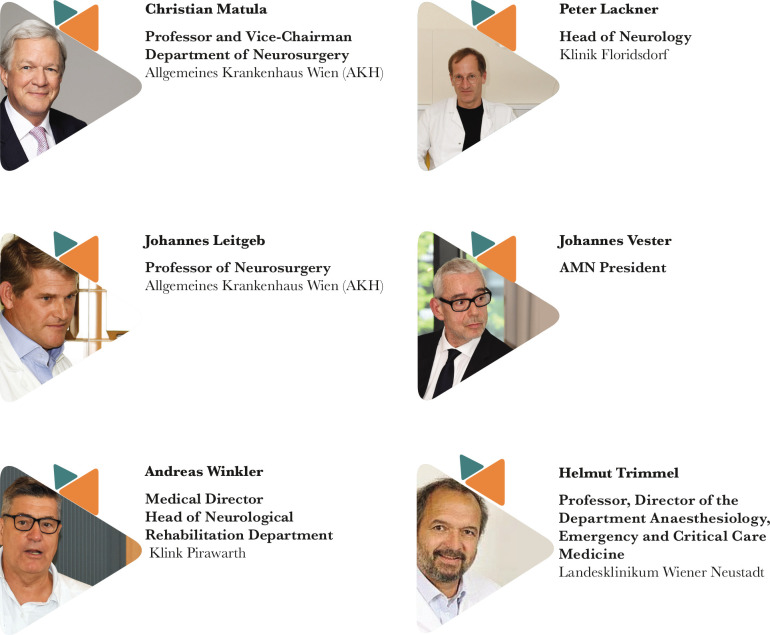


## SUMMARY OF THE EVENT

### Day 1

The first day of NTSC took place at Allgemeines Krankenhaus Wien (AKH), where each of the program tutors welcomed the participants and offered a brief presentation on their dedicated day of training. In the introduction, Prof. Christian Matula presented the objectives and goals of the NTSC Vienna, respectively the shift in the treatment paradigm of neurotrauma, from short-term focus to long-term follow-up. The introduction was followed by a self-presentation of each delegate focusing on their motivation to attend the NTSC and a country presentation providing insight on the epidemiology, treatment, and current issues in Traumatic Brain Injury (TBI).

Further on, Prof. Peter Lackner presented Klinik Floridsdorf, one of the newest and most modern hospitals in Vienna, the clinic that accommodates the Simulation Center where the last day of the NTSC would take place. Afterwards, Prof. Johannes Vester offered a comprehensive lecture on the “Multidimensional Approach in TBI Research”, showcasing the current situation of TBI clinical research and questions arising from clinical trials. Professor Vester summed up some of the lessons learned from experience in clinical research and pinpointed how a multidimensional approach would generate substantial savings by modern trial design. Lastly, Prof. Andreas Winkler presented Klinik Pirawarth, a rehabilitation clinic the participants would visit on the fourth day.

**Figure:**
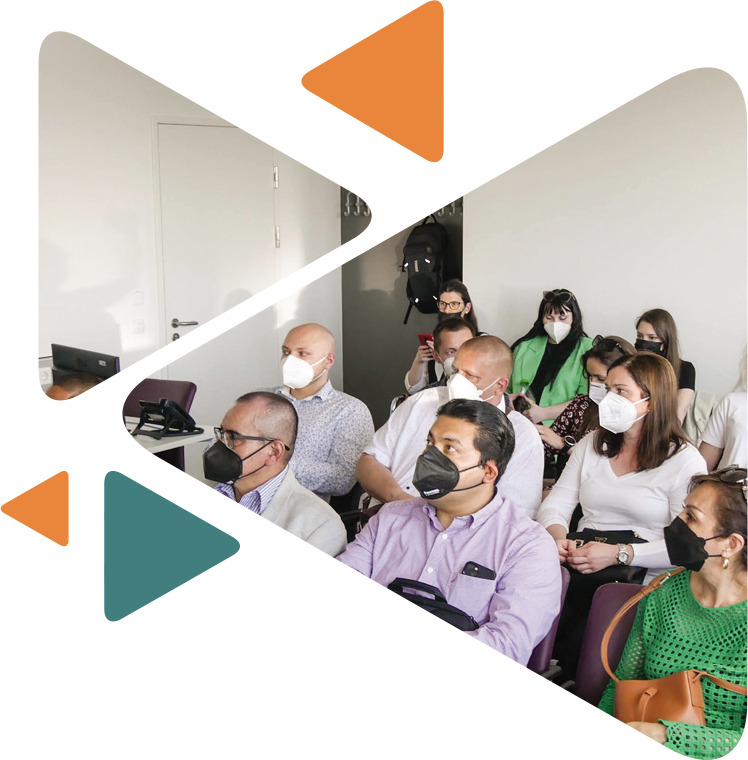


### Day 2

The second day of NTSC took place at Landesklinikum Wiener Neustadt, a teaching hospital of the Medical Universities of Vienna and Graz, as well as of the University of Applied Sciences Wiener Neustadt. The clinic is divided into 14 departments and five institutes and it serves as a specialized hospital for the surrounding area of Wiener Neustadt/OST. The airfield is the base for the air rescue center and it has several hangars: one for the Christophorus 3, one for the intensive care transport helicopter Christophorus ITH, also stationed here, and one for a backup helicopter.

The day started with OA Dr. Daniel Csomor who held a presentation on the *Air rescue system for primary and secondary missions*. His lecture began with the general outlines of prehospital emergency medicine, including emergency calls in Austria, dispatch center, emergency services and infrastructure, and continued with showcasing the numbers and roles of air rescue as part of the ÖAMTC (Austrian Automobile, Motorcycle and Touring Club). Dr. Csomor then presented the structure of an air rescue team composed of three staff members: the pilot, the emergency physician, and a paramedic, detailing the differences in the roles of the emergency physician and paramedic. Based on the presented studies, physician-staffed helicopter emergency medical service has a beneficial impact on both management of TBI patients and the emergency physicians with respect to the outcome of patients with severe TBI. The following section described the tasks of emergency physicians, from initial assessment to recommendations for management and diagnostics. In summary, Dr. Herzer underlined the importance of definitive treatment of patients with traumatic brain injury, a treatment that starts and is significantly influenced at the scene of the accident, with the following aims:

**Figure:**
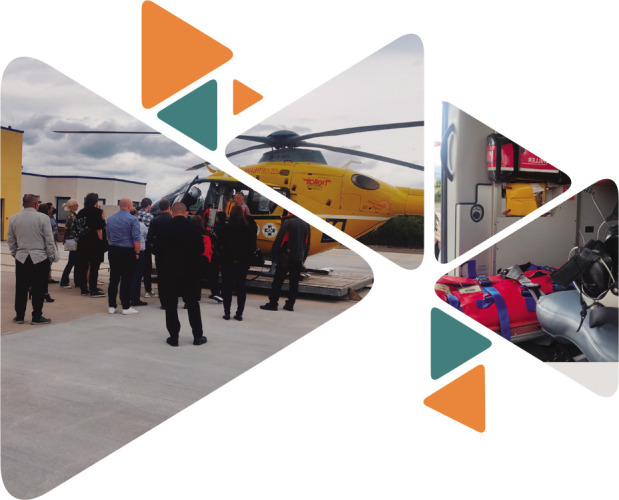

- Assessment of the state of consciousness and evaluation of injury pattern;
- Securing vital functions, breathing and circulation;
- Stabilization of cervical spine;
- Treatment of life-threatening associated injuries;
- Selection of the right destination hospital;
- Transport and documentation.

After the presentations, the participants had the chance to visit the airshed to explore and discuss about the equipment used in rescue missions. Dr. Daniel Csomor offered a full tour, shared details about the structure of the rescue team with each member's duties during a rescue action, and fully described the summer/winter equipment. The participants could take photos and ask for additional information.

On the second part of the day, participants and speakers moved to a meeting venue, where Prof. Johannes Vester lectured on the challenges and opportunities when publishing on neurotrauma. This presentation was a valuable learning opportunity, delivering insight on how to avoid heterogeneity in clinical trials by choosing ''the gentleman agreement'', the importance and impact of ''noise control'' in data (*e.g*., controlling weak motivation of staff, imprecise instructions, turnover, lack of standardization across sites, dropouts, missing data, imprecise instructions, deviations in schedule), and the pathway towards successful trial conduct. Prof. Vester also explained the typical reporting of Traumatic Brain Injury (TBI) in studies, and the issue of dichotomization of results, alongside the importance of multidimensional approaches, highlighting lessons from the CAPTAIN trials. This session also included an interactive activity with the participants: each country had to choose one specific issue and offer insights into the situation in their countries [see Table 1]. Dr. Iulia Vadan, neurologist, discussed the opportunities for dialogue and exchange of expertise among clinicians, researchers, and health professionals from all health systems, promoted by the AMN organization in the last couple of years. The next point of her presentation highlighted the disproportionality of the burden caused by traumatic brain injury in low- and middle-income countries compared to high-income countries, mainly due to health system organization and healthcare service delivery issues.

**Table 1:** Short insights into the obstacles in TBI research and information gap regarding TBI in NTSC participating countries.

Team	Question	Answer
**Egypt**	What is the main obstacle in your institution to performing clinical research on Brain Trauma?	The difficulties in communication and cooperation when working in teams The diversity of the encountered cases
**Mexico**		The lack of a TBI registry The fragmentation of the healthcare system, which follows a three-tier system, leading to difficulty in getting information
**The Philippines**	What is the main information gap regarding TBI in your country and what kind of publication could close it?	The geographical layout of the country, which consists of more than 7000 islands The very small number of doctors and an even smaller number of neurosurgeons scattered all over these islands, leading to lack of representation because patients can only receive services in the few big medical centers
**Poland**		Lack of data The need for standardized treatment procedures Work overload Communication issues between doctors
**Romania**	What is the main obstacle in your institution to performing clinical research on Brain Trauma?	Lack of motivation among staff members Communication problems among specialists, mainly due to the multiple sites scattered around the city and accommodating different specialities The availability of information, as databases only offer data from 2012 onward
**Slovakia**		The size of their hospital - small, with no neurosurgery department and no university affiliation

Dr. Vadan also introduced PRESENT (Patient REgistry – Short Essential NeuroTrauma), a TBI registry piloted in Cluj-Napoca, Romania, in 2022. She presented its mission, namely the collection of multidisciplinary, multidimensional, and longitudinal data from all care levels of traumatic brain injury on an international basis. The registry's vision is to serve as a low-threshold go-to hub for basic TBI data collection, both in countries with low access to data collection mechanisms as well as in more advanced healthcare settings. Its objectives focus on developing an evidence-informed record, offering e-accessibility and user-friendliness, collecting only essential data, and tailoring the instrument to the clinician's needs through intense pre-testing and piloting.

After the introduction of PRESENT, Dr. Iulia Vadan outlined the current status of this project in Romania, presenting the results from the pre-test (pilot) version of the registry on 32 TBI patients, which offered beneficial lessons for the next steps in the development of the registry. During the presentation, Dr. Vadan encouraged feedback from the participants on improving the registry. The participants exchanged impressions and actively got involved in discussions.

**Figure:**
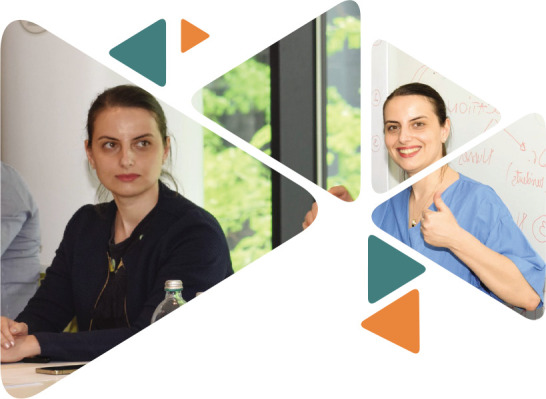


### Day 3

The third day of NTSC took place at Allgemeines Krankenhaus Wien (AKH) – Vienna General Hospital, starting with a short description of the care unit, presented by Prof. Christian Matula as a “city in a city” due to its large buildings with 30 different departments. The AKH employs more than 9,000 people, amongst them 1,603 physicians and about 4,500 allied health and nursing workers, treating over 100,000 patients per year.

After the introductory presentation, Prof. Karl Rossler, Head of the Neurosurgery Department, addressed a warm welcome to the participants and invited everyone to visit all the facilities in the building. He also talked about the excellent flow of their multidisciplinary teams, not only for neurotrauma but also for pediatric and neurosurgery departments.

The following presentation focused on Neurotrauma Management at the University Hospital of Vienna, a subject approached from three different perspectives. Prof. Klaus Klein presented the anesthesiologist's point of view, Prof. Johannes Leitgeb presented his viewpoint as a traumatologist, and the last perspective was that of Prof. Christian Matula, who presented the neurosurgeon's view.

The next activity of the day actively involved the participants divided into three small groups visiting the Trauma Operating Room, Emergency Room, and Simulation Room, according to a rotation plan. Similar activities continued under the supervision of Doctors Johannes Leitgeb, Wolfgang Machold and Anna Antoni, with case discussions on Emergency and Shock Room Simulation.

Based on the rotation plan, the groups, guided by Prof. Christian Matula, Dr. Phillip Dodler, and Dr. Arthur Hossmann, visited the Neurosurgery Unit, with each group covering main sections:

- Neurosurgery Operating Room (OR) and Intraoperative Monitoring (IOM) Areas;
- Magnetic Resonance Imaging (MRI) and Endovascular Treatment Area;
- Intracranial Pressure (ICP) Mapping and Micro Dialyzer.

Neurosurgery Simulation rotations continued with a *Neurotrauma Radiology Forum*, under the supervision of Dr. Ammer Mallouhi, followed by *Bone Flap Management* by Dr. Mathias Millesi. Interactive case discussions took place during the visits.

The very actual subject of Interdisciplinary Neurotrauma Management was again discussed from three different points of view *i.e*.:

- The anesthesiologist - Prof. Klaus Klein;
- The traumatologist - Prof. Johannes Leitgeb;
- The neurosurgeon - Prof. Christian Matula.

Professors Matula and Leitgeb then presented and expand- ed the idea of setting up a neurotrauma program and team. The participants were actively involved in the discussions, and the day ended with an open forum.

**Figure:**
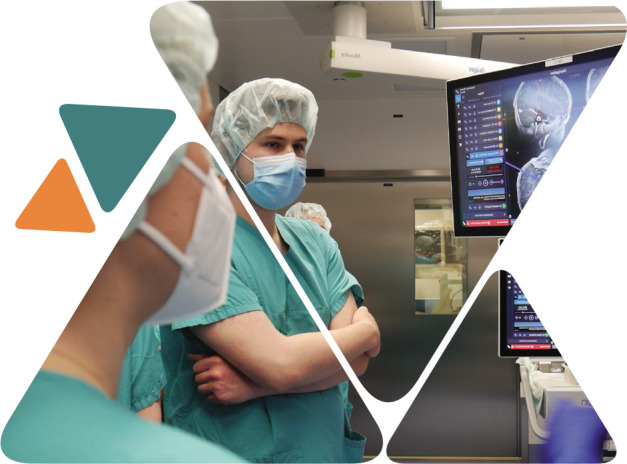


### Day 4

The 4^th^ day of NTSC took place at the Klinik Pirawarth, a rehabilitation clinic where the tutor of the day was Prof. Andreas Winkler.

Klinik Pirawarth is located in Lower Austria's wine district, 25 kilometers northeast of Vienna. The clinic has 360 beds and a 450 person team that cares for patients around the clock. A team of medical specialists, general practitioners, therapists, and nurses collaborate with patients in the clinic to develop an individual training program that includes creative and holistic approaches.

Professor Winkler started the day with an introduction to the program, where he pinpointed the historical development of neurorehabilitation, tasks and methods of rehabilitation, the challenges and opportunities of rehabilitation in the 21^st^ century, with a glimpse behind the scenes. The presentation covered general considerations regarding TBI, epidemiological aspects of neurotrauma, and the structural and functional architecture of TBI, as seen in imaging methods, such as Magnetic Resonance Imaging (MRI), and computed tomography (CT), Diffusion Tensor Imaging (DTI) and fiber tractography. Furthermore, he highlighted the leading diagnoses encountered at Pirawarth and discussed the Austrian insurance system.

**Figure:**
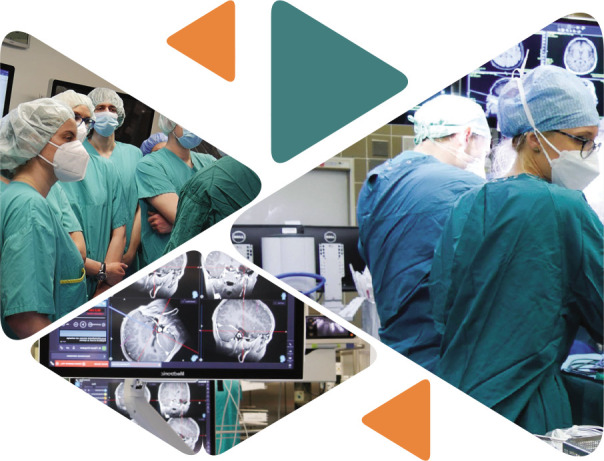


Moreover, the discussion covered aspects related to neurore- habilitation in the elderly population and insights into diagnostics, treatment, and appropriate supportive therapies.

The day continued with an inspiring presentation on the aspects of speech, language, and swallowing therapy in TBI patients by Andrea Harsanyi and Antje Bernas-Stiel, covering the disorders of speech and language, aspects related to swallowing and swallowing dysfunction, screening and examination. Followingly, a presentation entitled “Assessing and Promoting Motor control – PT E&ET” was conducted by Miriam Galoppi (MSc – PT) and Ines Schandl (MSc – OT) and was focused on sensorimotor deficits in the context of TBI, assessment and goal setting, as well as various aspects related to their treatment. The next presentation of the day covered the topic of neuro-psychological assessment and rehabilitation in TBI, by Mag. Brigit Brenner-Walter (Clinical Neuropsychologist), pinpointing a wide set of topics, from the specifics of neuropsychology in TBI, localization of TBI and neuropsychological profile, factors of influence, neuropsychological evaluation, assessment and screening, to the specific challenges in mild/moderate to severe TBI, and the reality of caregiver distress as well as its assessment. It also covered the topics of neuropsychological rehabilitation, reconstruction, compensation and training of functions, and coping strategies. The last presentation of the day targeted the domain of orthoptics, including information on the assessment and treatment paradigms.

The presentations were followed by hands-on experience targeting:

- *Spasticity, perception and electrotherapy* by I. Schandl, M. Galoppi, K. Grammelhofer, S. Pleil;
- *Swallowing Assessments* by A. Harsanyi, A. Bernas-Stiel;
- Selected neuropsychological assessment tools by B. Brenner-Walter;
- Orthoptic assessment and treatment by S. Muller.

The last workshop included insight into a fascinating and rarely encountered domain of the disorders of sight (*e.g*., visual acuity, visual field defects), their assessment and ways to be approached within the neurorehabilitation process.

### Day 5

The last day of NTSC took place at Klinik Floridsdorf, where Prof. Peter Lackner offered a glimpse into one of the most complex simulation centers worldwide, with a high-tech setup including complex mannequins that react to stimuli, and a top-class video and audio monitoring room.

The Floridsdorf Klinik was founded in 2018 as part of the Vienna Health Association. The 800-bed hospital combines the latest standards and the greatest possible comfort. The motto of the hospital is ʺpatient comes firstʺ and it represents an example of how the interplay of technology, architecture, and medicine can create a healthcare facility of the future, where people can heal and recover quickly.

The day started with an introduction by Prof. Karl Schebesta and a thoughtful discussion about the importance of multidisciplinarity and collaboration completed by a set of teamwork exercises.

**Figure:**
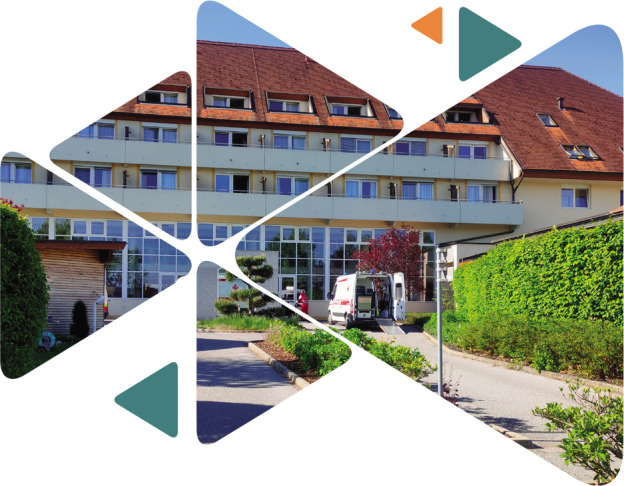


The participants were divided into two groups for the following activities of the day. While the first group participated in a training under the coordination of the simulation team, the other group followed and discussed the presentations of Prof. Lackner, working together on short presentations of country implementation plans.

The highlight of the day was the simulation of neurotrauma cases, where the participants had to actively collaborate to stabilize and treat the patient (mannequin). The training's central idea was to establish what to do in an emergency scenario and how to approach difficult situations as a leader or member of the team. Their main goal was to teach the participants how to communicate with each other and how to keep the team running smoothly.

The day was completed with Prof. Lackner's tour of the hospital where the participants got a glimpse into the Stroke Unit and Neurological Ward. An impressive feature of the Stroke Unit was the automatic electronic data collection by the medical monitoring equipment.

All the above reflect the importance of multimodal approaches to neurotrauma treatment, making the Neurotrauma Treatement Simulation Center (NTSC) a high-end training program for all specialists involved in the patient's pathway.

**Figure:**